# Antithrombotic therapy in adults with ectatic coronary artery disease: a systematic review and network meta-analysis

**DOI:** 10.1186/s43044-025-00612-8

**Published:** 2025-01-22

**Authors:** Alireza Azarboo, Mohammad Shahabaddin Daneshvar, Alireza Sattari Abroy, Ramin Assempoor, Aryan Taghvaei, Ali Nasrollahizadeh, Mohsen Hajiqasemi, Amirhossein Ghaseminejad-Raeini, Kaveh Hosseini

**Affiliations:** 1 Tehran Heart Center, Cardiovascular Diseases Research Institute, Tehran University of Medical Sciences, North Kargar Ave, Tehran, Iran; 2 Faculty of Medicine, Isfahan University of Medical Sciences, Isfahan, Iran; 3 Cardiac Primary Prevention Research Center, Cardiovascular Diseases Research Institute, Tehran University of Medical Sciences, Tehran, Iran; 4 School of Medicine, Tehran University of Medical Sciences, Tehran, Iran

**Keywords:** Coronary artery ectasia, Aspirin, DAPT, Anticoagulant, Major adverse cardiovascular events

## Abstract

**Background:**

Many studies have validated the use of antiplatelet or anticoagulant therapy in coronary artery ectasia (CAE) to reduce major adverse cardiovascular events (MACE); however, it is not completely known which group of these antithrombotic medications is more effective. The purpose of this systematic review and network meta-analysis was to evaluate the efficacy of different anti-thrombotic treatments in adult patients with CAE.

**Methods:**

This systematic review and meta-analysis followed preferred reporting items for systematic reviews and meta-analyses (PRISMA) guidelines as well as PRISMA extension statement for reporting of systematic reviews incorporating network meta-analyses and adhered to a registered predetermined methodology noted in the prospective register of systematic reviews (PROSPERO) protocol. Comprehensive searches were conducted until October 2024. Study selection, data extraction, and risk-of-bias assessments were independently performed by two reviewers. The pairwise meta-analysis compared the odds of MACE among patients receiving different antithrombotic therapies versus no treatment. The network meta-analysis (NMA) combined direct and indirect evidence to compare the efficacy of antithrombotic therapies for MACE.

**Results:**

Our systematic review included 5,039 adult patients suffering from CAE. The odds of MACE were higher in patients with no treatment when compared with those on dual antiplatelet therapy (DAPT) and aspirin monotherapy; although patients on anticoagulation demonstrated a lower incidence of MACE, the difference with the no treatment group did not reach statistical significance. Among various types of interventions in NMA, DAPT was the best in the treatment of CAE.

**Conclusions:**

Based on the surface under the cumulative ranking curve (SUCRA) value, DAPT is the most effective treatment in the prevention of MACE for CAE patients, followed by aspirin monotherapy and anticoagulant treatment.

**Supplementary Information:**

The online version contains supplementary material available at 10.1186/s43044-025-00612-8.

## Background

Coronary artery ectasia (CAE) is defined as the dilation of the coronary artery lumen [1]. The prevalence of CAE varies depending on the study setting and method, but it generally ranges from 0.3 to 5.3% in patients undergoing coronary angiography [2]. The exact etiology of CAE is not fully understood, but it may be due to genetic factors or acquired conditions. Possible etiologies include atherosclerosis, Kawasaki disease, mycotic or septic emboli, Marfan syndrome, arteritis from polyarteritis nodosa, Takayasu disease, systemic lupus erythematosus, or acute coronary syndrome (ACS) as one of paramount importance for CAE [3, 4]. Additionally, people with a higher body mass index (BMI), hypertension, high triglyceride levels, elevated levels of D-dimer, and males are at increased risk of developing CAE [5]. One of the most critical complications of CAE is thrombosis due to the ectatic vessel, which could lead to myocardial infarction [6]. ACS occurs due to the presence of aneurysmal segments of the coronary artery that produce sluggish or turbulent blood flow.

Managing CAE often poses a significant challenge due to the poorly understood mechanisms [7]. Patients with CAE often present with complex clinical profiles, including ACS, myocardial infarction, or angina; hence, they require specialized therapeutic strategies, including revascularization (e.g., percutaneous coronary intervention (PCI) and coronary artery bypass graft (CABG)) and medical therapy (e.g., beta-blockers, calcium channel blockers, and/or nitrates to manage symptoms such as angina). The choice of treatment for CAE depends on the size, location, and severity of the aneurysm, as well as the patient’s general condition and past medical history. Patients with CAE are at an increased risk of blood clotting and are often treated with anticoagulants (e.g., warfarin) and antiplatelets (e.g., aspirin, clopidogrel, and dipyridamole); however, it is not completely known which group of these antithrombotic medications is most effective [8].

Although a recent meta-analysis has explored the antithrombotic strategies in preventing coronary artery aneurysm (CAA) formation secondary to Kawasaki disease and the ensuing CAA cardiovascular complications in the pediatric population [4], there are very few direct head-to-head studies contrasting these treatments in our particular adult patient population. An extensive evaluation of the available data was necessary to fill this knowledge gap with the role of anti-thrombotic therapy in this population, and a network meta-analysis (NMA) was recommended to offer insights into the relative efficacy of different anti-thrombotic treatments in adult patients with CAE. Our study seeks to enhance clinical decision-making and patient outcomes by analyzing the available data on anti-thrombotic therapies for this particular subgroup of coronary artery disease (CAD) patients.

### Methods

We observed the "preferred reporting items for systematic reviews and meta-analyses" (the "PRISMA" statement)" in our meta-analysis as well as PRISMA extension statement for reporting of systematic reviews incorporating network meta-analyses of health care interventions [9]. This review observed the predetermined methodology noted in the prospective register of systematic reviews (PROSPERO) (CRD42023472561).

## Literature search strategy

A thorough search was conducted on electronic databases including PubMed, Scopus, and Web of Science. Due to extensive evolution of antithrombotic therapy in recent years, in order to prevent distortion of this meta-analysis results, we filtered our search from January 2014 to October 2024. The following keywords were implemented in our searches: ("coronary aneurysm" OR "CAE" OR "Coronary aneur*" OR "Coronary ecta*" OR "Coronary artery aneurysm") AND ("Anticoagulants" OR "Factor Xa Inhibitors" OR "Fibrin Modulating Agents" OR "Citric Acid" OR "Gabexate" OR "4-Hydroxycoumarins" OR "Heparinoids" OR "Dextrans" OR "Anticoagulation Bridge" OR "Sodium Citrate" OR "Heparin, Low-Molecular-Weight" OR "Anticoagulants" OR anticoagula* OR "anti-coagula*" OR antiplatelet*) AND ("Acute Coronary Syndrome" OR "Myocardial Infarction" OR "Non-ST Elevated Myocardial Infarction" OR "ST Elevation Myocardial Infarction" OR "Angina, Unstable" OR "Coronary Artery Disease" OR "Coronary Stenosis" OR "Coronary Disease" OR "cardiac infarct" OR "cardiac infarction"). The complete search strategy can be seen in supplementary file 1, Table S1. Additional eligible studies were sought by scouring the reference lists of the included studies. The studies were screened using a web-based tool for systematic review called Rayyan (https://www.rayyan.ai). Two reviewers (RA, AT) independently evaluated each article, and they also reviewed the full text and eliminated any duplicates. The inclusion–exclusion criteria were followed for selecting studies. The third author (SD) served as the moderator of consensus sessions to resolve any disagreements that might arise between reviewers.

## Inclusion and exclusion criteria

With “What is the comparative efficacy of anti-thrombotic therapies in adults with ectatic coronary artery disease?” as the study question, the studies that met the inclusion criteria were selected for analysis: 1) Participants: adults with coronary artery ectasia diagnosed via any available method; 2) Comparison: patients managed with single antiplatelet therapy (SAPT), dual antiplatelet therapy (DAPT), or anticoagulants; 3) Outcome: Major adverse cardiovascular events (MACE) defined as acute myocardial infarction (AMI), stroke, all-cause death, ACS, ischemic heart disease, heart failure, non-fatal re-infarction, and recurrent angina pain [9] (the result of each article that did not specifically mention MACE as an outcome was obtained by combining the constituent parts of MACE); 4) Types of the included studies: Observational and interventional studies. The following exclusion criteria were applied: 1) Conference abstracts, animal studies, expert-opinion studies, literature reviews, and case reports; 2) Studies including patients not treated with SAPT, DAPT, or anticoagulants; 3) Outdated articles i.e., before 2014 were also excluded.

## Data extraction and quality assessment

Two authors (AR, SD), following a comprehensive full-text screening, separately entered specified details into a standardized Excel spreadsheet: demographics, study details, hypertension prevalence, culprit artery, follow-up duration, outcome measures, and number of patients under the study's management of interest. The quality of studies was assessed using the 22-item Strengthening the Reporting of Observational Studies in Epidemiology (STROBE) checklist [10]. A STROBE score of 12 was considered high quality, and scores less than 12 were considered low quality [11]. The checklist for each study was filled out independently by two reviewers (AR, SD). Any discrepancies between the two reviewers were settled by consensus.

## Statistical analysis

R [version 4.4.2] was used to conduct data analysis in order to assess the MACE rates across distinct groups. Network meta-analysis (NMA) was achieved by combining direct and indirect evidence. Direct evidence refers to evidence obtained directly from studies, and indirect evidence refers to evidence obtained through one or more common comparators [12]. For example, in the absence of studies that directly compare anticoagulation and DAPT, anticoagulation and DAPT can be compared indirectly if both have been compared to no treatment intervention in studies. The OR compared the impact of various treatments on CAE. Fixed-effects network meta-analysis was utilized to estimate the relative impact of various interventions in multiple comparison studies. Effect sizes of the CAE were estimated by calculating OR and their corresponding 95% confidence intervals (CIs). The SUCRA value indicates the likelihood of each treatment being one of the best in the network, with higher values indicating better ranking probabilities. The ranking probabilities of each intervention were calculated using the SUCRA value and cumulative ranking plots. Consistency in NMA refers to the statistical concordance between direct and indirect comparisons. It is the numerical representation of transitivity within data [12]. Inconsistency in each pairwise comparison was evaluated utilizing inconsistency tests with the I^2^ statistic, where a p-value greater than 0.05 suggests consistency.

## Results

### Study selection and baseline characteristics

**Fig. 1:**
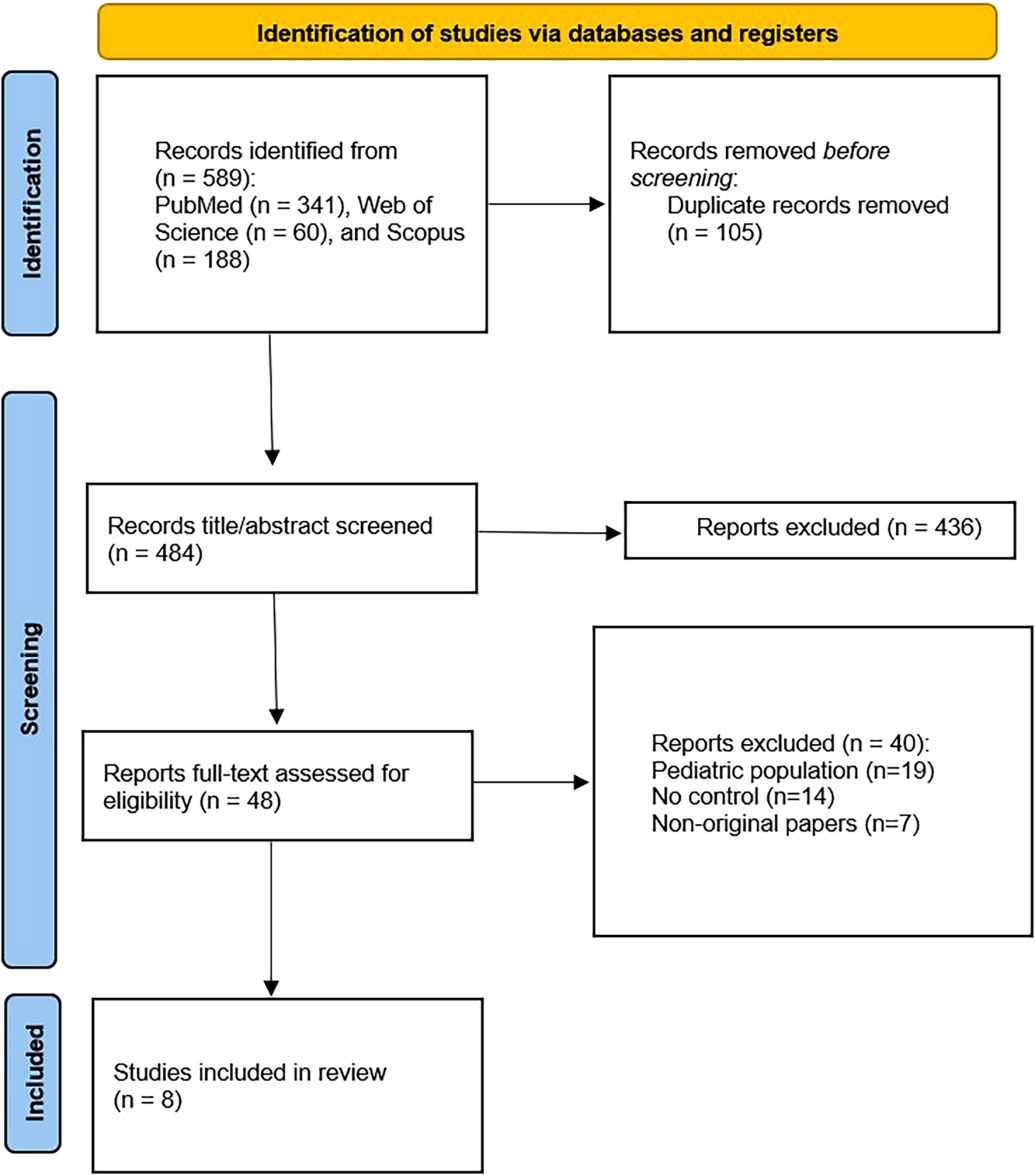
PRISMA flowchart of the study selection process

During the first systematic search of databases, a total of 589 studies were identified, with 105 duplicates subsequently eliminated. 484 records underwent title/abstract screening, of which 436 irrelevant studies were excluded. The term "irrelevant" refers to studies that, although retrieved due to the inclusion of search terms, were clearly outside the scope of the research. A total of 48 studies were assessed in full text; 8 met the inclusion criteria and were included in the systematic review; 6 of which reported sufficient data to be eligible for pairwise and network meta-analysis [2, 13–17] and the data of the other 2 that were incompatible with our analyses entered qualitative synthesis [18, 19] (Fig. 1).

The included studies were conducted in Australia [2], Spain [17], Japan [13], Korea [19], China [15], the USA [14], Singapore [18], and France [16]. The design of the studies was retrospective cohort [2, 13, 14, 16–19] and case–control [15]. Our meta-analysis included 5,039 adult patients suffering from CAE, with 82% of the population being male. The mean age of patients was 67.6 (range, 58.9–79.7). Also, 11.6 [17] and 116.4 [14] months constituted the minimum and maximum periods of follow-up among studies. The indication of coronary angiography procedure ranged from 32 to 100% ACS. Most studies selected MACE as the primary outcome for treatment assessment [2, 13–17, 19], in addition to thrombolysis in myocardial infarction (TIMI) flow [2, 18] and thrombus formation [18]. Treatment regimens included anticoagulation therapy [2, 13, 14, 16, 18], DAPT [2, 16, 18, 19], aspirin monotherapy [2, 15–17, 19], and no treatment [2, 13–17] (Table 1). Supplementary file 1, Table S2 summarizes the incidences of history of hypertension, diabetes mellitus, dyslipidemia, smoking, chronic renal failure, prior stroke, and family history of CAE in the included population. The culprit artery with the highest incidence of CAE was LAD (n = 1388) (Supplementary file 1, Table S3).

**Table 1:** Main baseline characteristics of the included studies

Study	Country	Design	Sample Size	ACS initial diagnosis	Age (mean ± SD)	Male (%)	Mean follow-up	Outcome	Treatment regimens	STROBE
Djohan (2022)	Singapore	Retrospective Cohort	1780	All STEMI	58.1 ± 12.2	87.3	36 months	length and diameter of aneurysmal or ectatic segment in the IRA, residual thrombus, post-procedure TIMI 3 flows	OAC, DAPT	15 (68%)
Doi (2017)	Japan	Retrospective Cohort	1698	All AMI	67.9 ± 12.1	71.4	49 months	MACE	Anticoagulation (target therapeutic range (%TTR) ≥ 60% under warfarin use), none (either sub optimally controlled %TTR (	17 (77.2%)
Gunasekaran (2019)	USA	Retrospective Cohort	634	24% UA 12% NSTEMI 8% STEMI	68.9 ± 11.5	79.4	116.4 months	MACE	Anticoagulation, DAPT, none	18 (81%)
Joo (2018)	Korea	Retrospective Cohort	347	49% UA 7% NSTEMI 8% STEMI	60.4 ± 9.8	66.9	38.8 months	MACE, nonfatal MI, stent thrombosis, or ISR	Aspirin monotherapy, DAPT (aspirin plus clopidogrel, aspirin plus cilostazol/ticlopidine), triple therapy	16 (72%)
Liang (2019)	China	Case–control	119	All patients had > 50% coronary artery stenosis	65 ± 11	70.6	NA	MACE	Aspirin monotherapy, none	19 (86%)
Matta (2023)	France	Retrospective Cohort	100	32% ACS	64.9 ± 12.6	82	46.2 months	MACE	Anticoagulation, aspirin monotherapy, DAPT, none	20 (91%)
Núñez-Gil (2018)	Spain	Retrospective Cohort	256	19% UA 32% NSTEMI 10% STEMI	65.6 ± 12.4	86.3	11.6 months	MACE	Anticoagulation, aspirin monotherapy, none	17 (77.2%)
Shanmugam (2017)	Australia	Retrospective Cohort	105	All STEMI	55.6 ± 11.2	95.2	36.6 months	MACE (long -term composite event rates), TIMI 3 flow, stenting	Anticoagulation, dual blood-thinning therapy, aspirin monotherapy, none	18 (81%)

*ACS*, Acute coronary syndrome; *AMI*, Acute myocardial infarction; *DAPT*, Dual antiplatelet therapy; *IRA*, Infarct-related artery; *ISR*, In-stent restenosis; *MACE*, Major adverse cardiac events; *MI*, Myocardial infarction; *NSTEMI*, Non-St-elevation myocardial infarction; *OAC*, Oral anticoagulation; *STEMI*, ST-Elevation myocardial infarction; *STROBE*, Strengthening the reporting of observational studies in epidemiology; *TIMI*, Thrombolysis in myocardial infarction; *TTR*, Time in therapeutic range; *UA*, Unstable angina

### Quantitative data synthesis

Due to substantial variations in the reported outcomes and treatment regimens across studies, we divided our meta-analysis into the following:

## Pairwise meta-analysis

**Fig. 2:**
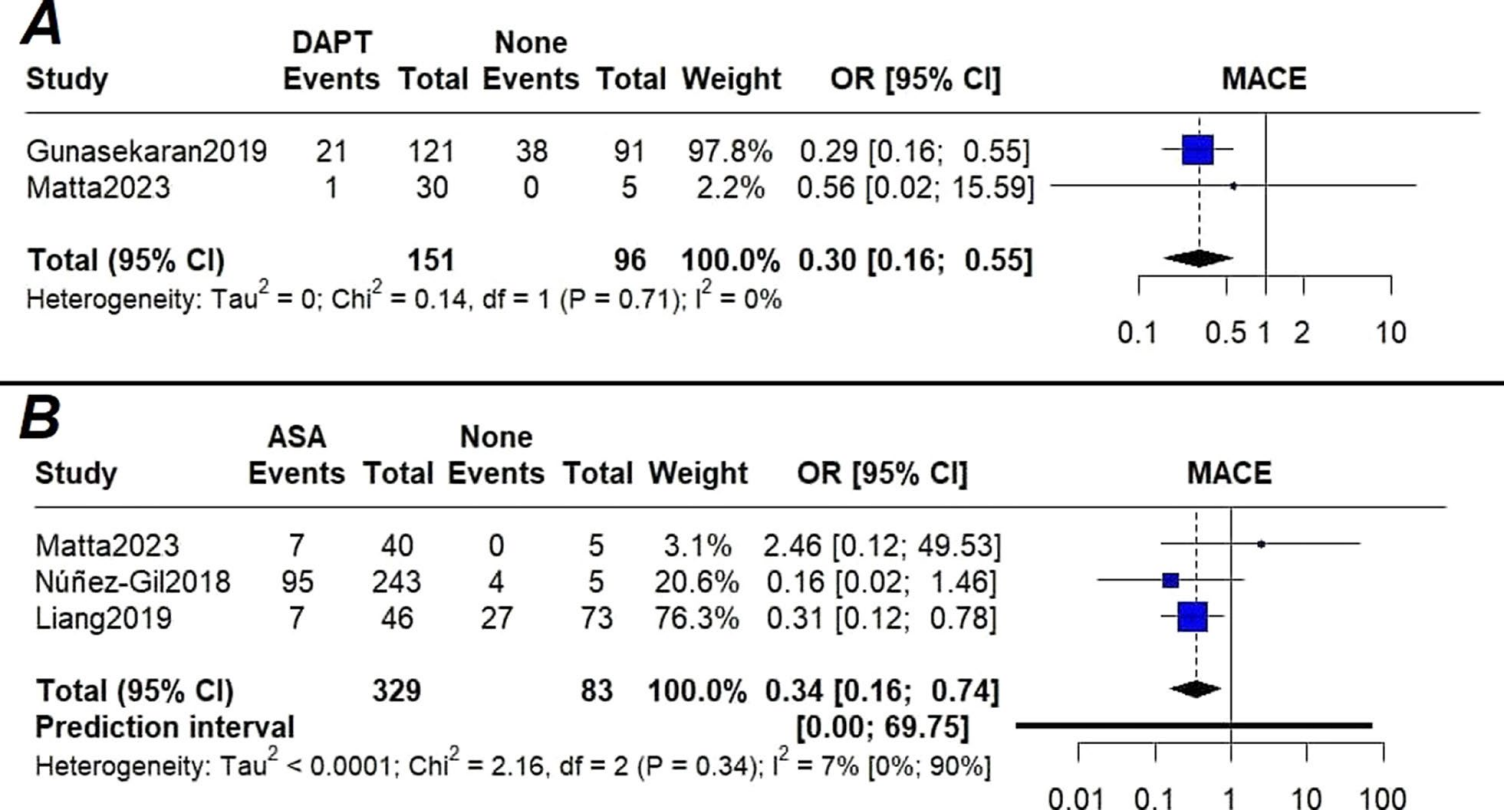
**A** Forest plot of the association of MACE with DAPT versus none; **B** Forest plot of the association of MACE with aspirin monotherapy versus none

**Fig. 3:**
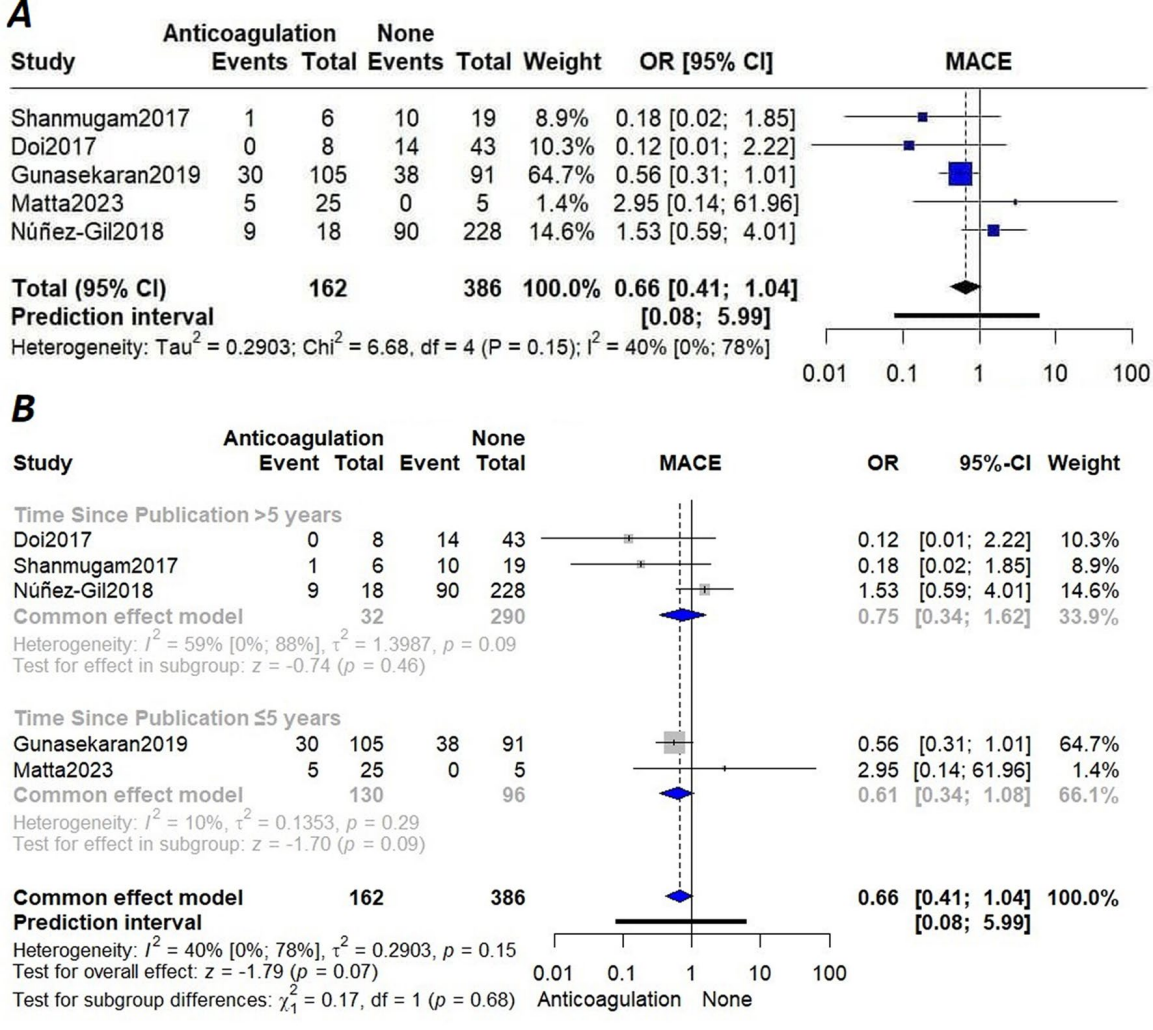
**A** Forest plot of the association of MACE with anticoagulation therapy versus none; **B** Subgroup analysis based on time since publication year of the association of MACE with anticoagulation therapy versus none

We performed a pairwise meta-analysis on patients under different treatments vs. those with no treatment. The odds of MACE were higher in patients with no treatment (labeled “None” in the figure) when compared with those on DAPT (OR[95%CI] = 0.30[0.16,0.55]; I^2^ = 0%) (Fig. 2A) and aspirin monotherapy (OR[95%CI] = 0.34[0.16,0.74]; I2 = 7.8%) (Fig. 2B); although patients on anticoagulation demonstrated a lower incidence of MACE, the difference with the no treatment group did not reach statistical significance (OR[95%CI] = 0.66[0.41,1.04]; I^2^ = 40.4%) (Fig. 3A). Subgroup analysis based on the publication year did not show any deviation from the overall finding (Fig. 3B).

## Network meta-analysis

**Fig. 4:**
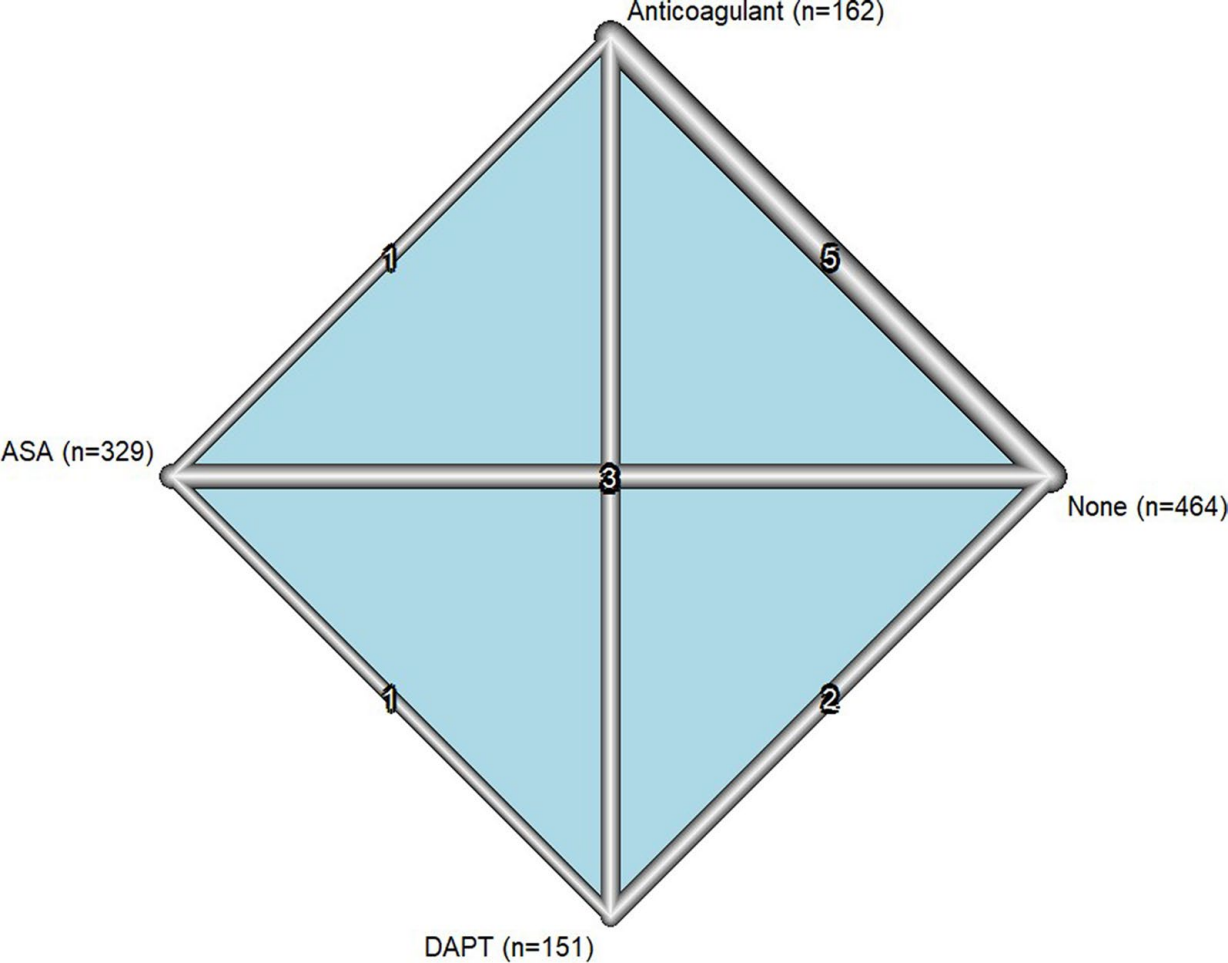
Network meta-analysis of eligible comparisons for MACE

**Fig. 5:**
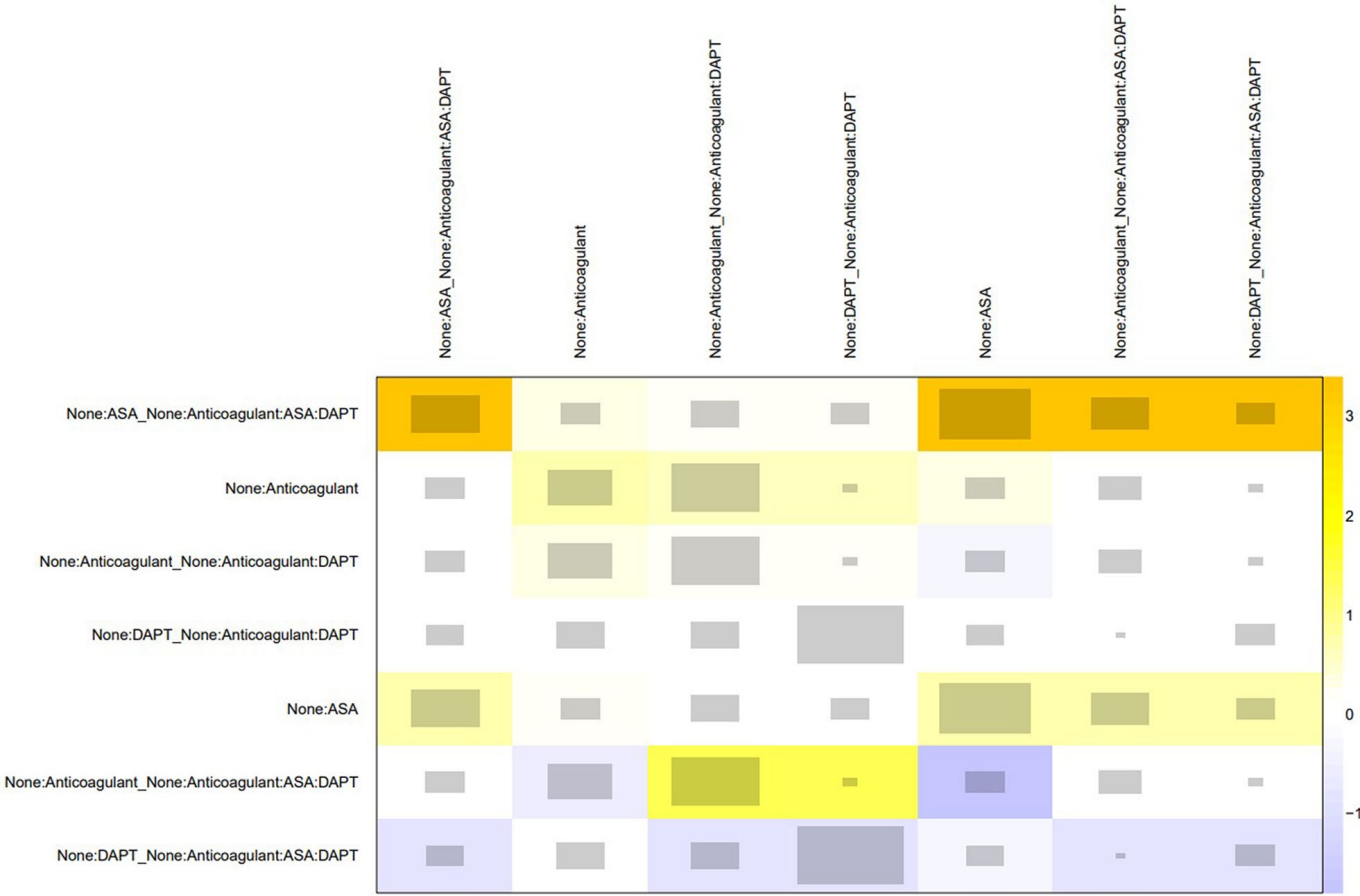
Network heat plot of eligible comparisons for MACE

**Fig. 6:**
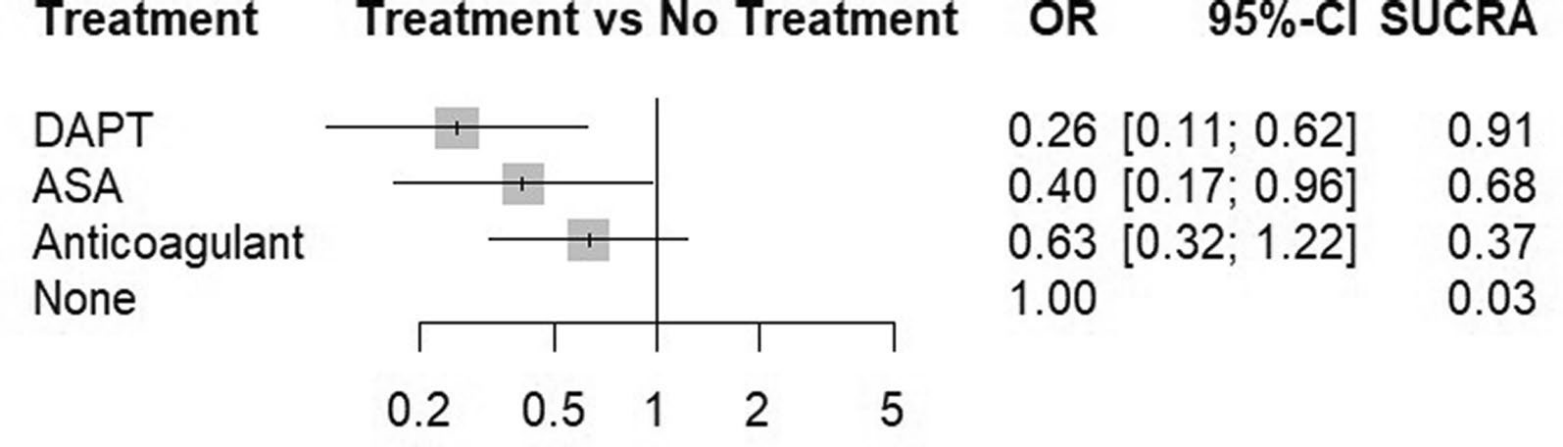
The surface under the cumulative ranking curve (SUCRA) ranking chart for MACE

**Fig. 7:**
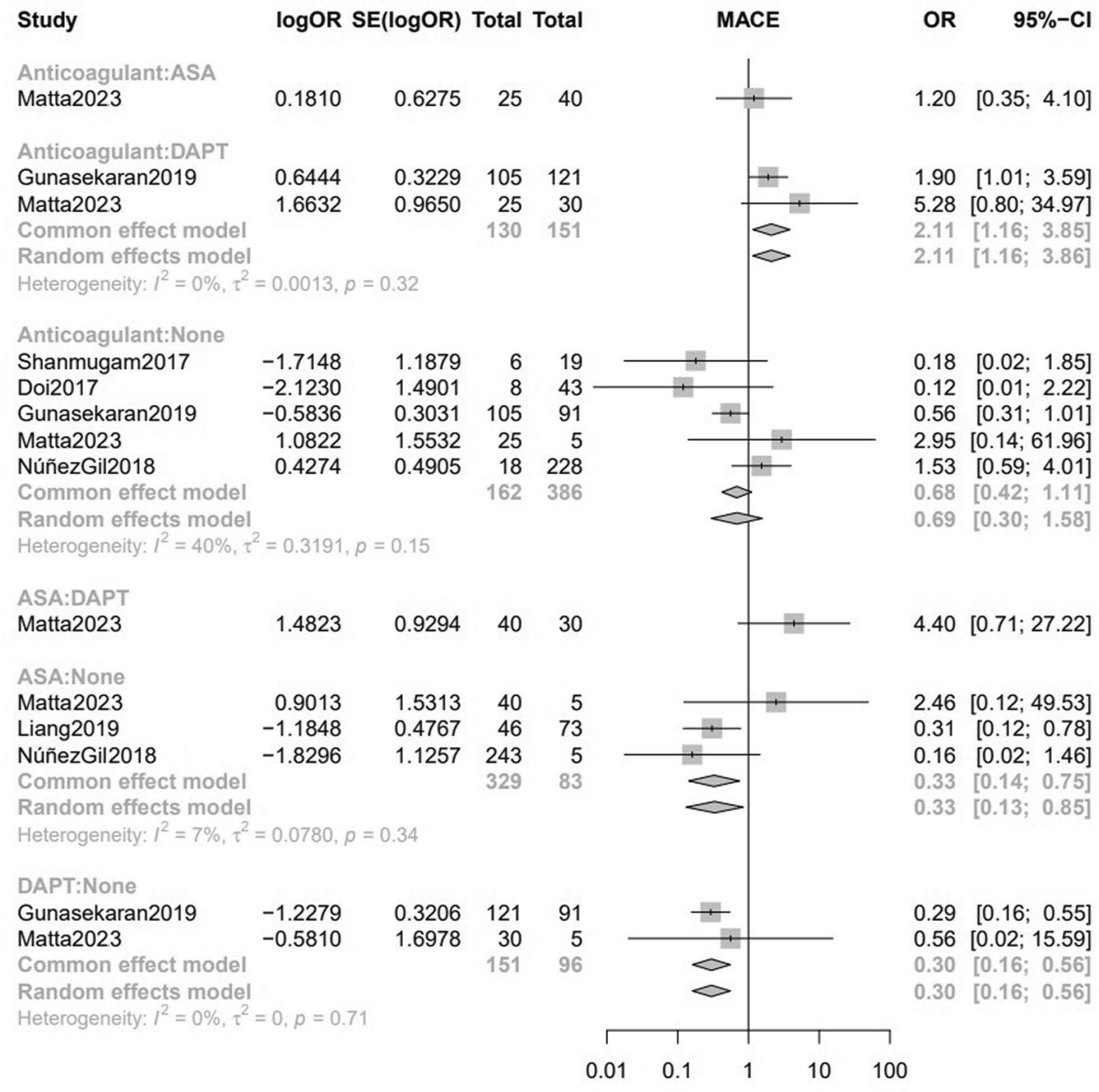
Head-to-head odds ratios intercomparisons of different interventions

Five studies [2, 13, 14, 16, 17] examined the incidence of MACE between 162 patients on anticoagulant treatment and 386 on no treatment. Three studies [15–17] with 329 aspirin patients and 83 patients on no treatment, as well as two studies with 151 DAPT patients and 96 patients on no treatment, evaluated the incidence of MACE (Fig. 4). Direct and indirect estimates (Supplementary File 1, Figure S1) were calculated via fixed-effect model due to low heterogeneity (I^2^ = 26.3%, *P* = 0.16). Based on the inconsistency test, no inconsistency was detected (*P* value = 0.35) (Fig. 5). Among various types of interventions, based on 1000 simulations, DAPT (SUCRA = 0.91) was the best in the treatment of CAE (OR [95%CI] = 0.26[0.11, 0.62]), followed by aspirin monotherapy (SUCRA = 0.68) (OR [95%CI] = 0.40[0.17, 0.96]) and anticoagulant therapy (SUCRA = 0.63) (OR [95%CI] = 0.63[0.32, 1.22]) (Fig. 6). Table 2 illustrates the league table with OR estimates of each pair of interventions and 95% CI for MACE outcomes. Head-to-head odds ratios intercomparisons of different interventions are also shown (Fig. 7).

**Table 2:** League table with OR estimates of each pair of interventions and 95% CI

**DAPT**	0.23 (0.04–1.40)	0.47 (0.26–0.86)	0.30 (0.16–0.56)
0.72 (0.31–1.66)	**ASA**	0.83 (0.24–2.85)	0.33 (0.14–0.75)
0.45 (0.25–0.79)	0.62 (0.29–1.32)	**Anticoagulant**	0.68 (0.42–1.11)
0.28 (0.16–0.51)	0.40 (0.20–0.80)	0.64 (0.40–1.01)	**None**

The bold diagonal line refers to the treatments

## Risk of bias and publication bias

STROBE scores for the included studies are shown in Table 1. The mean score percent ranged from 68 to 91% out of 22 items, showing a relatively low risk of bias. In pairwise meta-analysis, Egger’s test and funnel plot symmetry were utilized to assess the publication bias of the network meta-analysis. No publication bias was detected (*P* value = 0.82) (Supplementary file 1, Figure S2).

### Qualitative data synthesis

According to Matta et al. [16], there was no statistically significant difference in MACE-free survival favoring oral anticoagulant recipients (*P* = 0.557).

Shanmugam et al. [2] discovered that the majority of patients with EIRA were prescribed dual antiplatelet therapy upon discharge: 76.0% received aspirin combined with an ADP antagonist, 8.3% were on an ADP antagonist with warfarin, and 16.7% were on triple therapy including aspirin, ADP antagonist, and warfarin. Further examination of the procedural features of the EIRA group, focusing on warfarin usage, indicated that there were trends towards lower rates of stenting and TIMI 3 flow in patients with warfarin prescriptions compared to those without, although these trends were not statistically significant (33.3% vs. 63.2%, *P* = 0.34 for stenting and 16.7% vs. 57.9%, *P* = 0.20 for TIMI 3 flow). While the difference may not be statistically significant due to the low number of patients, it is worth mentioning that EIRA patients discharged on warfarin had a long-term clinical event rate of only 16.7%, whereas those not discharged on warfarin had a rate of 52.6%. In terms of treatment duration, just over 50% of EIRA patients were prescribed additional blood-thinning medication throughout the entire follow-up period, while 45.8% were only given aspirin for the entire 12 months. In individuals taking both blood-thinning medications for an extended period, we observed similar long-term composite event rates to those taking aspirin alone after the initial year, with no significant statistical variance (23.1% vs. 36.4%, *P* = 0.66).

Djohan et al. [18] reported that when compared against DAPT alone (length median (IQR) 41.7(16.5, 81.1), diameter median (IQR) 5.1(4.1, 6.3)) with *P* = 0.029 and *P* = 0.002, respectively, there was a significant association between length (median (IQR) 75.2(23.1, 115.6) and diameter (median (IQR) 7.4(6.1, 8.7) of aneurysmal or ectatic segment in the EIRA and discharge prescription of anticoagulation. The choice to administer oral anticoagulation was not influenced by the existence of post-procedure TIMI 3 flow (OAC% = 61.5 vs. DAPT alone% = 73.9; *P* = 0.45) or residual thrombus (OAC% = 46.2 vs. DAPT alone% = 39.1; *P* = 0.7).

Joo et al. [19] observed that of the 21 cases (26.9%) of MACE in the CAE group, 10 cases (12.8%) occurred under DAPT and 8 cases (10.3%) happened under aspirin monotherapy. MACE and DAPT maintenance did not significantly correlate. While aspirin monotherapy was responsible for two deaths in the CAE group, there was no significant correlation found between DAPT maintenance and the development of nonfatal MI, stent thrombosis, or ISR. Furthermore, there was no discernible variation in MACE based on the DAPT type. Thirteen patients receiving triple treatment had four MACEs (28.6%), thirty-three patients receiving aspirin plus clopidogrel had eight MACEs (24.2%), and eight patients receiving aspirin plus cilostazol/ticlopidine had five MACEs (27.8%).

## Discussion

In this systematic review and network meta-analysis, we evaluated the effectiveness of anti-thrombotic therapy in adults with ectatic coronary artery disease. Our findings shed light on the clinical outcomes associated with various treatment regimens and provide valuable insights into the management of this complex condition. In this study, we reviewed 8 eligible articles concerning CAE and their sequelae in adult patients; with a total of 5,039 patients. Our main interest was to compare MACE in those who received medical therapy, either aspirin, DAPT, or anticoagulants, with those who did not. We first compared patients receiving each therapy with non-treated patients through meta-analysis and then investigated all possible direct or indirect connections between different therapies through NMA.

CAE is linked to major adverse cardiac events by a pathophysiological mechanism involving coronary slow flow, which results in sluggish or turbulent blood flow. MACE increases the incidence of typical exercise-induced angina pectoris, myocardial infarction, thrombotic events, and sudden cardiac death [13, 20].

First, disturbance of the internal elastic laminal layer and chronic vascular inflammation are the main causes of inflammation linked to CAE, which in turn causes a decrease in medial elastic tissue. Furthermore, elevated levels of inflammatory biomarkers, including vascular endothelial growth factor, homocysteine, and C-reactive protein, have been associated with CAE, indicating a potential involvement in neovascularization and inflammation. So much so that all inflammatory biomarkers under investigation of a related meta-analysis were found higher in coronary artery ectasia patients vs. healthy controls (NLR: SMD [95%CI] = 0.73[0.27–1.20], hs-CRP: SMD [95%CI] = 0.96[0.64–1.28], IL-6: SMD [95%CI] = 2.68[0.95–4.41], TNF-a: SMD [95%CI] = 0.50[0.24–0.75], RDW: SMD [95%CI] = 0.56[0.26–0.87]) [21]. This inflammatory environment increases the risk of acute coronary events, such as myocardial infarction and ischemic stroke, by facilitating the development of atherosclerotic plaques and encouraging the production of thrombus. Furthermore, dysregulated matrix metalloproteinase activity exacerbates the sensitivity to plaque rupture and thrombotic events by further impairing the artery wall's structural integrity [22]. Studies show that CAE raises the levels of beta-TG, PF4, and plasma P-selectin, which in turn enhances platelet activation [23]. Therefore, many studies have validated the use of antiplatelet or anticoagulant therapy to reduce major adverse cardiovascular events [13, 15, 24].

DAPT typically comprises aspirin and a P2Y12 receptor inhibitor such as clopidogrel, prasugrel, or ticagrelor. The antiplatelet actions of DAPT have a pathophysiological role in preventing MACE in patients with CAE. By suppressing thromboxane A2, a powerful platelet aggregator, DAPT inhibits the enzyme cyclooxygenase-1, which lowers platelet aggregation and the development of blood clots in the arteries. This mechanism lowers the incidence of thrombotic events in the ectatic coronary arteries, which is essential in avoiding MACE, such as myocardial infarction and stroke, in patients with CAE [25]. A meta-analysis revealed that aspirin lowered the incidence of MACE, with shorter-term trials (≤ 5 years) showing a larger benefit than longer-term studies (> 5 years) [26]. Additionally, aspirin reduces inflammation by preventing the production of prostaglandins, mediators of inflammation. Aspirin serves to ameliorate endothelial dysfunction and slow the course of atherosclerosis, a chronic inflammatory condition marked by the deposition of lipid-rich plaques inside the artery walls, by modifying the inflammatory response within the arterial wall. This anti-inflammatory effect is especially important for CAE patients because inflammation has a role in the development and course of coronary artery ectasia [27]. The result of our meta-analysis, backing up the aforementioned mechanisms, showed that any type of antiplatelet treatment led to a decrease in MACE incidence; patients receiving aspirin therapy or DAPT had significantly lower rates of MACE compared to non-treated patients. Nevertheless, other healthcare aspects should be factored in when prescribing DAPT and aspirin. That the use of more recent P2Y12 receptor inhibitors may result in increased expenses, even if DAPT provides improved platelet inhibition and possible therapeutic advantages over aspirin monotherapy in CAE, especially in high-risk patients [28]. Although there is a higher risk of bleeding, data from larger populations with coronary artery disease indicates that DAPT may lower the incidence of MACE, including myocardial infarction and stent thrombosis [29]. Overall, the choice between aspirin monotherapy and DAPT should consider the balance between ischemic protection and bleeding risk, as well as patient preferences and healthcare resource utilization. Based on the surface under the cumulative ranking curve (SUCRA) value, we also found that DAPT is the most effective treatment for CAE, followed by aspirin and anticoagulant treatment.

Anticoagulants such as warfarin and heparin have been taken into consideration in the treatment of CAE because ectatic coronary arteries are prone to thrombosis, dissection, and spasm. Anticoagulant therapy has been suggested as a secondary preventive measure for CAE patients to avoid thrombus development [8]. Furthermore, a systematic review of case reports revealed that anticoagulants, in addition to either single or dual antiplatelet medication, may help lower the chance of ACS recurrence in patients with CAE [30]. In clinical practice, there are a number of possible drawbacks to anticoagulant therapy in CAE that need to be carefully examined. First of all, patients with CAE who may already be at risk for bleeding due to the existence of ectatic arteries are more susceptible to bleeding consequences from anticoagulants, including major bleeding events that can result in significant morbidity and mortality [30]. Furthermore, prothrombin time (PT), the international normalized ratio (INR), or activated partial thromboplastin time (aPTT) must be closely monitored when using anticoagulants. This can be difficult for patients and healthcare professionals and raise the possibility of medication errors [31]. Moreover, anticoagulants may have negative pharmacological interactions with other prescription drugs and dietary supplements, which calls for cautious management [32]. Anticoagulant use over an extended period of time may be linked to higher healthcare expenses and patient non-adherence, especially if regular monitoring and dose modifications are necessary [33]. Anticoagulants should generally be used with extreme caution in CAE, even if they may be beneficial in some clinical situations, especially since we found treatment with anticoagulants had no statistically or clinically significant difference regarding MACE in comparison to non-treated patients.

## Future recommendations

Future research should focus on conducting well-designed, large-scale, randomized controlled trials specifically in patients with CAE. These trials should directly compare various anti-thrombotic regimens to elucidate the most effective and safest treatment strategy. Furthermore, research on newer and wider varieties of antiplatelet and anticoagulant agents in different dosages, such as direct oral anticoagulants, in CAE is warranted.

## Limitations

Our meta-analysis had some limitations. First, a significant number of the included studies are retrospective in character, which presents biases and limitations typical of retrospective data collection and analysis. Furthermore, no randomized clinical trials have been conducted so far, particularly RCTs, to assess the advantages and potential complications of these therapies thoroughly. Second, different follow-up timeframes across studies led to heterogeneity in our results since different follow-up periods could influence the occurrence of outcomes, i.e., MACE. Third, the fact that the number of included studies was limited prevented us from conducting further sensitivity analyses. It is also important to assess the safety outcomes of the different treatments, such as bleeding or thrombosis, for clinical decision-making that was not feasible in this meta-analysis. Fourth, due to the lack of clarification and subgrouping by the included studies, umbrella terms such as DAPT, MACE, and anticoagulant therapy pose a core limitation of the interpretation of our results.

## Conclusions

Based on our meta-analysis and network meta-analysis, treatment with aspirin and DAPT can significantly decrease the incidence rate for MACE. DAPT, when compared to other medical therapies through NMA, showed a major advantage in lowering MACE incidence. Aspirin therapy and anticoagulant therapy were second and third on the list, respectively. It is considerable that all three kinds of therapies can decrease MACE incidence to some extent, although more studies are needed to be certain about these conclusions.

## Supplementary Information

Additional file1.

## Data Availability

The data that support the findings of this study are available from the corresponding author (K.H) upon reasonable request.

## Abbreviations

ACS: Acute coronary syndrome
DP: Adenosine diphosphate
AMI: Acute myocardial infarction
ASA: Acetylsalicylic acid (aspirin)
BMI: Body mass index
CABG: Coronary artery bypass graft
CAD: Coronary artery disease
CAA: Coronary artery aneurysm
CAE: Coronary artery ectasia
CI: Confidence interval
DAPT: Dual antiplatelet therapy
IQR: Interquartile range
LAD: Left anterior descending (artery)
MACE: Major adverse cardiovascular events
NMA: Network meta-analysis
OR: Odds ratio
PCI: Percutaneous coronary intervention
PRISMA: Preferred reporting items for systematic reviews and meta-analyses
PROSPERO: Prospective register of systematic reviews
SMD: Standardized mean difference
STROBE: Strengthening the reporting of observational studies in epidemiology
SUCRA: Surface under the cumulative ranking curve
TIMI: Thrombolysis in myocardial infarction
VEGF: Vascular endothelial growth factor
