# Supplementary material for: Antithrombotic therapy in adults with ectatic coronary artery disease: a systematic review and network meta-analysis
Source: Egypt Heart J. 2025 Jan 22;77:13. doi: 10.1186/s43044-025-00612-8 (PMC11754575; doi:10.1186/s43044-025-00612-8)
Supplement: Supplementary file 1 — Additional file1. [file 43044_2025_612_MOESM1_ESM.docx]

| Table S1. Search strategy for each database | | |
| --- | --- | --- |
| Database | Search Query | Results |
| PubMed | ("coronary aneurysm"[Mesh] OR "CAE"[tiab] OR "Coronary aneur*"[tiab] OR "Coronary ecta*"[tiab] OR "Coronary artery aneurysm"[tiab] OR "Coronary artery ecta*"[tiab] OR "Aneurysm, Coronary"[tiab] OR "Aneurysms, Coronary"[tiab] OR "Coronary Aneurysms"[tiab] OR ("Coronary Vessels"[Mesh] AND ("aneur*"[tiab] OR "ecta*"[tiab]))) AND (("Anticoagulants"[Mesh] OR "Anticoagulants" [Pharmacological Action] OR "Factor Xa Inhibitors"[Mesh] OR "Fibrin Modulating Agents"[Mesh] OR "Citric Acid"[Mesh] OR "Gabexate"[Mesh] OR "4-Hydroxycoumarins"[Mesh] OR "Heparinoids"[Mesh] OR "Dextrans"[Mesh] OR "Anticoagulation Bridge"[Mesh] OR "Sodium Citrate"[Mesh] OR "Heparin, Low-Molecular-Weight"[Mesh] OR "Anticoagulants"[Mesh] OR anticoagula*[tiab] OR "anti-coagula*"[tiab] OR antiplatelet*[tiab] OR "anti-platelet*"[tiab] OR antithrombocytic*[tiab] OR "anti-thrombocytic"[tiab] OR antithrombotic*[tiab] OR "anti-thrombotic*"[tiab] OR antithrombin*[tiab] OR "anti-thrombin*"[tiab] OR "vitamin K antagonist*"[tiab] OR ((thromb*[tiab] OR platelet*[tiab] OR glycoprotein*[tiab] OR "blood clot*"[tiab] OR "vitamin k"[tiab] OR "vitamin-k"[tiab] OR "factor xa"[tiab]) AND (antagonist*[tiab] OR inhibit*[tiab])) OR antistenocardin[tiab] OR cortab[tiab] OR curantil[tiab] OR curantyl[tiab] OR efosin[tiab] OR germed[tiab] OR kurantil[tiab] OR persantin*[tiab] OR prandial[tiab] OR prexin[tiab] OR solantin[tiab] OR antistasin[tiab] OR apixaban[tiab] OR betrixaban[tiab] OR darexaban[tiab] OR edoxaban[tiab] OR eribaxaban[tiab] OR fidexaban[tiab] OR fondaparinux[tiab] OR idrabiotaparinux[tiab] OR idraparinux[tiab] OR letaxaban[tiab] OR otamixaban[tiab] OR razaxaban[tiab] OR rivaroxaban[tiab] OR tanogitran[tiab] OR xarelto[tiab] OR yagin[tiab] OR argatroban[tiab] OR bivalirudin[tiab] OR bothrojaracin[tiab] OR dabigatran[tiab] OR desulfatohirudin[tiab] OR efegatran[tiab] OR flovagatran[tiab] OR hirudin[tiab] OR hirugen[tiab] OR inogatran[tiab] OR lepirudin[tiab] OR melagatran[tiab] OR napsagatran[tiab] OR odiparcil[tiab] OR pegmusirudin[tiab] OR tanogitran[tiab] OR ximelagatran[tiab] OR phenprocoumon[tiab] OR falithrom[tiab] OR falithrome[tiab] OR fenprocoumon[tiab] OR liquamar[tiab] OR marcoumar[tiab] OR marcumar[tiab] OR phenprocouman[tiab] OR phenprocoumarol[tiab] OR phenprocoumon[tiab] OR phenprocumarol[tiab] OR acenocoumarin[tiab] OR acenocoumarine[tiab] OR acenocoumarole[tiab] OR acenocoumarolum[tiab] OR acenocumarol[tiab] OR acenocumarolo[tiab] OR acenokumarin[tiab] OR acitrom[tiab] OR neo sintrom[tiab] OR neosintrom[tiab] OR neositron[tiab] OR nicoumalone[tiab] OR nicumalon[tiab] OR nitrowarfarin[tiab] OR sincoumar[tiab] OR sincumar[tiab] OR sinkumar[tiab] OR sinthrom[tiab] OR sinthrome[tiab] OR sintrom[tiab] OR sintroma[tiab] OR sintron[tiab] OR syncoumar[tiab] OR syncumar[tiab] OR syntrom[tiab] OR trombostop[tiab] OR zotil[tiab] OR brodifacoum[tiab] OR coumatetralyl[tiab] OR dicoumarol[tiab] OR tioclomarol[tiab] OR "alpha-heparin"[tiab] OR clarin[tiab] OR eleparon[tiab] OR elheparin[tiab] OR epiheparin[tiab] OR "hep lock"[tiab] OR hepalean[tiab] OR heparin*[tiab] OR heparina[tiab] OR heparine[tiab] OR "heparinic acid"[tiab] OR hepcon[tiab] OR "lipo hepin"[tiab] OR lipohepin[tiab] OR "lipo hepin"[tiab] OR lipocortin[tiab] OR liquaemin[tiab] OR liquemin[tiab] OR liquemine[tiab] OR menaven[tiab] OR monoparin[tiab] OR multiparin[tiab] OR panheprin[tiab] OR pularin[tiab] OR "thrombo vetren"[tiab] OR thromboliquine[tiab] OR thrombophob[tiab] OR thrombosamine[tiab] OR vetren[tiab] OR vister[tiab] OR adomiparin[tiab] OR antixarin[tiab] OR ardeparin[tiab] OR bemiparin[tiab] OR certoparin[tiab] OR dalteparin[tiab] OR danaparoid[tiab] OR deligoparin[tiab] OR dociparstat[tiab] OR embolex[tiab] OR enoxaparin[tiab] OR fondaparinux[tiab] OR hirudoid[tiab] OR idrabiotaparinux[tiab] OR idraparinux[tiab] OR nadroparin[tiab] OR necuparanib[tiab] OR parnaparin[tiab] OR reviparin[tiab] OR semuloparin[tiab] OR suleparoide[tiab] OR tedelparin[tiab] OR tinzaparin[tiab]) OR (antiplatelet[mesh] OR "Platelet Aggregation Inhibitors"[Mesh] OR aspirin*[tiab] OR "acetylsalicylic acid"[tiab] OR "acetyl salicylate"[tiab] OR "acetyl salicylic acid"[tiab] OR "acetylic salicylic acid"[tiab] OR "acetylsalicylic acid"[tiab] OR "acetylosalicylic acid"[tiab] OR acetylsal[tiab] OR "acetylsalicyclic acid"[tiab] OR acenterine[tiab] OR acesal[tiab] OR acetan[tiab] OR acetard[tiab] OR aceticyl[tiab] OR acetilum[tiab] OR acetonyl[tiab] OR acetophen[tiab] OR acetosal[tiab] OR "acetosalicylic acid"[tiab] OR acetosalin[tiab] OR acetylin[tiab] OR acetylo[tiab] OR acetylon[tiab] OR acetylsalicyl[tiab] OR acetylsalicylate[tiab] OR acetysal[tiab] OR acidulatum[tiab] OR "acidum acetylsalicylicum"[tiab] OR acylpyrin[tiab] OR acylpyrine[tiab] OR adiro[tiab] OR alabukun[tiab] OR anopyrin[tiab] OR ansin[tiab] OR aptor[tiab] OR asaphen[tiab] OR aspec[tiab] OR aspent[tiab] OR aspergum[tiab] OR aspex[tiab] OR aspisol[tiab] OR asteric[tiab] OR astrix[tiab] OR bamyl[tiab] OR biprin[tiab] OR boxazin[tiab] OR caprin[tiab] OR cardioaspirina[tiab] OR cartia[tiab] OR caspirin[tiab] OR catalgine[tiab] OR catalgix[tiab] OR claradin[tiab] OR colfarit[tiab] OR contrheuma[tiab] OR dispirin[tiab] OR dolean[tiab] OR durlaza[tiab] OR dusil[tiab] OR easprin[tiab] OR ecasil[tiab] OR ecosprin[tiab] OR ecotrin[tiab] OR empirin[tiab] OR endosprin[tiab] OR entericin[tiab] OR enterosarine[tiab] OR entrophen[tiab] OR extren[tiab] OR globentyl[tiab] OR godamed[tiab] OR helicon[tiab] OR idotyl[tiab] OR istopirin[tiab] OR magnecyl[tiab] OR measurin[tiab] OR melabon[tiab] OR micristin[tiab] OR mikristin[tiab] OR naspro[tiab] OR "nu-seal*"[tiab] OR nuseals[tiab] OR ostoprin[tiab] OR paracin[tiab] OR pengo[tiab] OR polopiryna[tiab] OR polopirin[tiab] OR premaspin[tiab] OR reumyl[tiab] OR rhodine[tiab] OR rhonal[tiab] OR ronal[tiab] OR salacetin[tiab] OR saletin[tiab] OR sedergine[tiab] OR "sodium acetylsalicylate"[tiab] OR solpyron[tiab] OR solprin[tiab] OR solupsa*[tiab] OR tapal[tiab] OR temagin[tiab] OR tromalyt[tiab] OR turivital[tiab] OR vazalore[tiab] OR verin[tiab] OR vitalink[tiab] OR xaxa[tiab] OR zorprin[tiab] OR ajoene[tiab] OR aloxiprin[tiab] OR anagrelide[tiab] OR ancrod[tiab] OR applaggin[tiab] OR aprosulate[tiab] OR aspalatone[tiab] OR ataprost[tiab] OR atopaxar[tiab] OR beraprost[tiab] OR brilinta[tiab] OR brilique[tiab] OR cangrelor[tiab] OR caplacizumab[tiab] OR cilostazol[tiab] OR clopidogrel[tiab] OR clopilet[tiab] OR cicaprost[tiab] OR ciprostene[tiab] OR cryptolepine[tiab] OR dazoxiben[tiab] OR dehydrocilostazol[tiab] OR dermatan[tiab] OR dextran*[tiab] OR dipyridamole[tiab] OR elinogrel[tiab] OR "enfenamic acid"[tiab] OR esuberaprost[tiab] OR glenzocimab[tiab] OR ghilanten[tiab] OR ibustrin[tiab] OR iloprost*[tiab] OR ifetroban[tiab] OR imolamine[tiab] OR indobufen[tiab] OR isbogrel[tiab] OR iscover[tiab] OR itazigrel[tiab] OR linotroban[tiab] OR lixazinone[tiab] OR nafazatrom[tiab] OR naxaprostene[tiab] OR octimibate[tiab] OR oxagrelate[tiab] OR pamicogrel[tiab] OR "pentosan polysulfate*"[tiab] OR picotamide[tiab] OR piracetam[tiab] OR plafibride[tiab] OR plavix[tiab] OR possia[tiab] OR prasugrel[tiab] OR prostacyclin[tiab] OR pentoxifyllin*[tiab] OR prostacyclin[tiab] OR prasugrel*[tiab] OR rafigrelide[tiab] OR samixogrel[tiab] OR sarpogrelate[tiab] OR satigrel[tiab] OR selatogrel[tiab] OR sulfinpyrazone[tiab] OR taprostene[tiab] OR terbogrel[tiab] OR terutroban[tiab] OR triflusal[tiab] OR ticagrelor[tiab] OR ticlopidine[tiab] OR tretoquinol[tiab] OR uproleselan[tiab] OR abciximab[tiab] OR aggrastat[tiab] OR albolabrin[tiab] OR arginylglycylaspartylserine[tiab] OR bitistatin[tiab] OR contortrostatin[tiab] OR disintegrin*[tiab] OR echistatin[tiab] OR elarofiban[tiab] OR eptifibatid*[tiab] OR fradafiban[tiab] OR gantofiban[tiab] OR glycylarginylglycylaspartylserine[tiab] OR integrilin kistrin[tiab] OR lamifiban[tiab] OR lefradafiban[tiab] OR lotrafiban[tiab] OR orbofiban[tiab] OR "repro"[tiab] OR roxifiban[tiab] OR sibrafiban[tiab] OR tirofiban[tiab] OR triflavin[tiab] OR trigamin[tiab] OR xemilofiban[tiab] OR annexin[tiab] OR caplacizumab[tiab] OR effient[tiab] OR efient[tiab] OR prasugrel[tiab])) AND ("Acute Coronary Syndrome"[Mesh] OR "Myocardial Infarction"[Mesh] OR "Non-ST Elevated Myocardial Infarction"[Mesh] OR "ST Elevation Myocardial Infarction"[Mesh] OR "Angina, Unstable"[Mesh] OR "Coronary Artery Disease"[Mesh] OR "Coronary Stenosis"[Mesh] OR "Coronary Disease"[Mesh] OR "cardiac infarct"[tiab] OR "cardiac infarction"[tiab] OR "cardial infarct"[tiab] OR "heart attack"[tiab] OR "heart infarct"[tiab] OR "heart infarction"[tiab] OR "heart micro infarction"[tiab] OR "heart muscle infarction"[tiab] OR "infarction, heart"[tiab] OR "myocardial infarct"[tiab] OR "myocardial infarction"[tiab] OR "myocardium infarct"[tiab] OR "myocardium infarction"[tiab] OR "second heart attack"[tiab] OR "subendocardial infarction"[tiab] OR "transmural cardiac infarction"[tiab] OR "transmural heart infarction"[tiab] OR "transmural infarction, heart"[tiab] OR "acute coronary syndrome"[tiab] OR "acute coronary syndromes"[tiab] OR acs[tiab] OR ami[tiab] OR "st elevated mi"[tiab] OR "st elevated myocardial infarction"[tiab] OR "st elevation mi"[tiab] OR "st elevation myocardial infarction"[tiab] OR "st segment elevated myocardial infarction"[tiab] OR "st segment elevation mi"[tiab] OR "st segment elevation heart infarction"[tiab] OR "st segment elevation myocardial infarction"[tiab] OR stemi[tiab] OR nstemi[tiab] OR "non st elevated mi"[tiab] OR "non st elevated myocardial infarction"[tiab] OR "non st elevation mi"[tiab] OR "non st elevation myocardial infarction"[tiab] OR "non st segment elevated myocardial infarction"[tiab] OR "non st segment elevation mi"[tiab] OR "non st segment elevation heart infarction"[tiab] OR "non st segment elevation myocardial infarction"[tiab] OR "non stemi"[tiab] OR "non-st elevated myocardial infarction"[tiab] OR "angina pectoris, unstable"[tiab] OR "angina, unstable"[tiab] OR "unstable angina"[tiab] OR "unstable angina pectoris"[tiab] OR uap[tiab]) AND ("Adult"[Mesh] OR adult*[tiab] OR man[tiab] OR men[tiab] OR woman[tiab] OR women[tiab]) | 341 |
| Web of Science | TS=("coronary aneurysm" OR "CAE" OR "Coronary aneur*" OR "Coronary ecta*" OR "Coronary artery aneurysm" OR "Coronary artery ecta*" OR "Aneurysm, Coronary" OR "Aneurysms, Coronary" OR "Coronary Aneurysms" OR ("Coronary Vessels" AND ("aneur*" OR "ecta*" ))) AND TS= (("Anticoagulants" OR "Anticoagulants" [Pharmacological Action] OR "Factor Xa Inhibitors" OR "Fibrin Modulating Agents" OR "Citric Acid" OR "Gabexate" OR "4-Hydroxycoumarins" OR "Heparinoids" OR "Dextrans" OR "Anticoagulation Bridge" OR "Sodium Citrate" OR "Heparin, Low-Molecular-Weight" OR "Anticoagulants" OR anticoagula* OR "anti-coagula*" OR antiplatelet* OR "anti-platelet*" OR antithrombocytic* OR "anti-thrombocytic" OR antithrombotic* OR "anti-thrombotic*" OR antithrombin* OR "anti-thrombin*" OR "vitamin K antagonist*" OR ((thromb* OR platelet* OR glycoprotein* OR "blood clot*" OR "vitamin k" OR "vitamin-k" OR "factor xa" ) AND (antagonist* OR inhibit* )) OR antistenocardin OR cortab OR curantil OR curantyl OR efosin OR germed OR kurantil OR persantin* OR prandial OR prexin OR solantin OR antistasin OR apixaban OR betrixaban OR darexaban OR edoxaban OR eribaxaban OR fidexaban OR fondaparinux OR idrabiotaparinux OR idraparinux OR letaxaban OR otamixaban OR razaxaban OR rivaroxaban OR tanogitran OR xarelto OR yagin OR argatroban OR bivalirudin OR bothrojaracin OR dabigatran OR desulfatohirudin OR efegatran OR flovagatran OR hirudin OR hirugen OR inogatran OR lepirudin OR melagatran OR napsagatran OR odiparcil OR pegmusirudin OR tanogitran OR ximelagatran OR phenprocoumon OR falithrom OR falithrome OR fenprocoumon OR liquamar OR marcoumar OR marcumar OR phenprocouman OR phenprocoumarol OR phenprocoumon OR phenprocumarol OR acenocoumarin OR acenocoumarine OR acenocoumarole OR acenocoumarolum OR acenocumarol OR acenocumarolo OR acenokumarin OR acitrom OR neo sintrom OR neosintrom OR neositron OR nicoumalone OR nicumalon OR nitrowarfarin OR sincoumar OR sincumar OR sinkumar OR sinthrom OR sinthrome OR sintrom OR sintroma OR sintron OR syncoumar OR syncumar OR syntrom OR trombostop OR zotil OR brodifacoum OR coumatetralyl OR dicoumarol OR tioclomarol OR "alpha-heparin" OR clarin OR eleparon OR elheparin OR epiheparin OR "hep lock" OR hepalean OR heparin* OR heparina OR heparine OR "heparinic acid" OR hepcon OR "lipo hepin" OR lipohepin OR "lipo hepin" OR lipocortin OR liquaemin OR liquemin OR liquemine OR menaven OR monoparin OR multiparin OR panheprin OR pularin OR "thrombo vetren" OR thromboliquine OR thrombophob OR thrombosamine OR vetren OR vister OR adomiparin OR antixarin OR ardeparin OR bemiparin OR certoparin OR dalteparin OR danaparoid OR deligoparin OR dociparstat OR embolex OR enoxaparin OR fondaparinux OR hirudoid OR idrabiotaparinux OR idraparinux OR nadroparin OR necuparanib OR parnaparin OR reviparin OR semuloparin OR suleparoide OR tedelparin OR tinzaparin ) OR (antiplatelet OR "Platelet Aggregation Inhibitors" OR aspirin* OR "acetylsalicylic acid" OR "acetyl salicylate" OR "acetyl salicylic acid" OR "acetylic salicylic acid" OR "acetylsalicylic acid" OR "acetylosalicylic acid" OR acetylsal OR "acetylsalicyclic acid" OR acenterine OR acesal OR acetan OR acetard OR aceticyl OR acetilum OR acetonyl OR acetophen OR acetosal OR "acetosalicylic acid" OR acetosalin OR acetylin OR acetylo OR acetylon OR acetylsalicyl OR acetylsalicylate OR acetysal OR acidulatum OR "acidum acetylsalicylicum" OR acylpyrin OR acylpyrine OR adiro OR alabukun OR anopyrin OR ansin OR aptor OR asaphen OR aspec OR aspent OR aspergum OR aspex OR aspisol OR asteric OR astrix OR bamyl OR biprin OR boxazin OR caprin OR cardioaspirina OR cartia OR caspirin OR catalgine OR catalgix OR claradin OR colfarit OR contrheuma OR dispirin OR dolean OR durlaza OR dusil OR easprin OR ecasil OR ecosprin OR ecotrin OR empirin OR endosprin OR entericin OR enterosarine OR entrophen OR extren OR globentyl OR godamed OR helicon OR idotyl OR istopirin OR magnecyl OR measurin OR melabon OR micristin OR mikristin OR naspro OR "nu-seal*" OR nuseals OR ostoprin OR paracin OR pengo OR polopiryna OR polopirin OR premaspin OR reumyl OR rhodine OR rhonal OR ronal OR salacetin OR saletin OR sedergine OR "sodium acetylsalicylate" OR solpyron OR solprin OR solupsa* OR tapal OR temagin OR tromalyt OR turivital OR vazalore OR verin OR vitalink OR xaxa OR zorprin OR ajoene OR aloxiprin OR anagrelide OR ancrod OR applaggin OR aprosulate OR aspalatone OR ataprost OR atopaxar OR beraprost OR brilinta OR brilique OR cangrelor OR caplacizumab OR cilostazol OR clopidogrel OR clopilet OR cicaprost OR ciprostene OR cryptolepine OR dazoxiben OR dehydrocilostazol OR dermatan OR dextran* OR dipyridamole OR elinogrel OR "enfenamic acid" OR esuberaprost OR glenzocimab OR ghilanten OR ibustrin OR iloprost* OR ifetroban OR imolamine OR indobufen OR isbogrel OR iscover OR itazigrel OR linotroban OR lixazinone OR nafazatrom OR naxaprostene OR octimibate OR oxagrelate OR pamicogrel OR "pentosan polysulfate*" OR picotamide OR piracetam OR plafibride OR plavix OR possia OR prasugrel OR prostacyclin OR pentoxifyllin* OR prostacyclin OR prasugrel* OR rafigrelide OR samixogrel OR sarpogrelate OR satigrel OR selatogrel OR sulfinpyrazone OR taprostene OR terbogrel OR terutroban OR triflusal OR ticagrelor OR ticlopidine OR tretoquinol OR uproleselan OR abciximab OR aggrastat OR albolabrin OR arginylglycylaspartylserine OR bitistatin OR contortrostatin OR disintegrin* OR echistatin OR elarofiban OR eptifibatid* OR fradafiban OR gantofiban OR glycylarginylglycylaspartylserine OR integrilin kistrin OR lamifiban OR lefradafiban OR lotrafiban OR orbofiban OR "repro" OR roxifiban OR sibrafiban OR tirofiban OR triflavin OR trigamin OR xemilofiban OR annexin OR caplacizumab OR effient OR efient OR prasugrel )) AND TS= ("Acute Coronary Syndrome" OR "Myocardial Infarction" OR "Non-ST Elevated Myocardial Infarction" OR "ST Elevation Myocardial Infarction" OR "Angina, Unstable" OR "Coronary Artery Disease" OR "Coronary Stenosis" OR "Coronary Disease" OR "cardiac infarct" OR "cardiac infarction" OR "cardial infarct" OR "heart attack" OR "heart infarct" OR "heart infarction" OR "heart micro infarction" OR "heart muscle infarction" OR "infarction, heart" OR "myocardial infarct" OR "myocardial infarction" OR "myocardium infarct" OR "myocardium infarction" OR "second heart attack" OR "subendocardial infarction" OR "transmural cardiac infarction" OR "transmural heart infarction" OR "transmural infarction, heart" OR "acute coronary syndrome" OR "acute coronary syndromes" OR acs OR ami OR "st elevated mi" OR "st elevated myocardial infarction" OR "st elevation mi" OR "st elevation myocardial infarction" OR "st segment elevated myocardial infarction" OR "st segment elevation mi" OR "st segment elevation heart infarction" OR "st segment elevation myocardial infarction" OR stemi OR nstemi OR "non st elevated mi" OR "non st elevated myocardial infarction" OR "non st elevation mi" OR "non st elevation myocardial infarction" OR "non st segment elevated myocardial infarction" OR "non st segment elevation mi" OR "non st segment elevation heart infarction" OR "non st segment elevation myocardial infarction" OR "non stemi" OR "non-st elevated myocardial infarction" OR "angina pectoris, unstable" OR "angina, unstable" OR "unstable angina" OR "unstable angina pectoris" OR uap ) AND TS= ("Adult" OR adult* OR man OR men OR woman OR women ) | 60 |
| Scopus | ( TITLE-ABS-KEY ( "coronary aneurysm" OR "CAE" OR "Coronary aneur*" OR "Coronary ecta*" OR "Coronary artery aneurysm" OR "Coronary artery ecta*" OR "Aneurysm, Coronary" OR "Aneurysms, Coronary" OR "Coronary Aneurysms" OR ( "Coronary Vessels" AND ( "aneur*" OR "ecta*" ) ) ) ) AND ( TITLE-ABS-KEY ( "Anticoagulants" OR "Factor Xa Inhibitors" OR "Fibrin Modulating Agents" OR "Citric Acid" OR "Gabexate" OR "4-Hydroxycoumarins" OR "Heparinoids" OR "Dextrans" OR "Anticoagulation Bridge" OR "Sodium Citrate" OR "Heparin, Low-Molecular-Weight" OR "Anticoagulants" OR anticoagula* OR "anti-coagula*" OR antiplatelet* OR "anti-platelet*" OR antithrombocytic* OR "anti-thrombocytic" OR antithrombotic* OR "anti-thrombotic*" OR antithrombin* OR "anti-thrombin*" OR "vitamin K antagonist*" OR ( ( thromb* OR platelet* OR glycoprotein* OR "blood clot*" OR "vitamin k" OR "vitamin-k" OR "factor xa" ) AND ( antagonist* OR inhibit* ) ) OR antistenocardin OR cortab OR curantil OR curantyl OR efosin OR germed OR kurantil OR persantin* OR prandial OR prexin OR solantin OR antistasin OR apixaban OR betrixaban OR darexaban OR edoxaban OR eribaxaban OR fidexaban OR fondaparinux OR idrabiotaparinux OR idraparinux OR letaxaban OR otamixaban OR razaxaban OR rivaroxaban OR tanogitran OR xarelto OR yagin OR argatroban OR bivalirudin OR bothrojaracin OR dabigatran OR desulfatohirudin OR efegatran OR flovagatran OR hirudin OR hirugen OR inogatran OR lepirudin OR melagatran OR napsagatran OR odiparcil OR pegmusirudin OR tanogitran OR ximelagatran OR phenprocoumon OR falithrom OR falithrome OR fenprocoumon OR liquamar OR marcoumar OR marcumar OR phenprocouman OR phenprocoumarol OR phenprocoumon OR phenprocumarol OR acenocoumarin OR acenocoumarine OR acenocoumarole OR acenocoumarolum OR acenocumarol OR acenocumarolo OR acenokumarin OR acitrom OR neo AND sintrom OR neosintrom OR neositron OR nicoumalone OR nicumalon OR nitrowarfarin OR sincoumar OR sincumar OR sinkumar OR sinthrom OR sinthrome OR sintrom OR sintroma OR sintron OR syncoumar OR syncumar OR syntrom OR trombostop OR zotil OR brodifacoum OR coumatetralyl OR dicoumarol OR tioclomarol OR "alpha-heparin" OR clarin OR eleparon OR elheparin OR epiheparin OR "hep lock" OR hepalean OR heparin* OR heparina OR heparine OR "heparinic acid" OR hepcon OR "lipo hepin" OR lipohepin OR "lipo hepin" OR lipocortin OR liquaemin OR liquemin OR liquemine OR menaven OR monoparin OR multiparin OR panheprin OR pularin OR "thrombo vetren" OR thromboliquine OR thrombophob OR thrombosamine OR vetren OR vister OR adomiparin OR antixarin OR ardeparin OR bemiparin OR certoparin OR dalteparin OR danaparoid OR deligoparin OR dociparstat OR embolex OR enoxaparin OR fondaparinux OR hirudoid OR idrabiotaparinux OR idraparinux OR nadroparin OR necuparanib OR parnaparin OR reviparin OR semuloparin OR suleparoide OR tedelparin OR tinzaparin ) OR ( antiplatelet OR "Platelet Aggregation Inhibitors" OR aspirin* OR "acetylsalicylic acid" OR "acetyl salicylate" OR "acetyl salicylic acid" OR "acetylic salicylic acid" OR "acetylsalicylic acid" OR "acetylosalicylic acid" OR acetylsal OR "acetylsalicyclic acid" OR acenterine OR acesal OR acetan OR acetard OR aceticyl OR acetilum OR acetonyl OR acetophen OR acetosal OR "acetosalicylic acid" OR acetosalin OR acetylin OR acetylo OR acetylon OR acetylsalicyl OR acetylsalicylate OR acetysal OR acidulatum OR "acidum acetylsalicylicum" OR acylpyrin OR acylpyrine OR adiro OR alabukun OR anopyrin OR ansin OR aptor OR asaphen OR aspec OR aspent OR aspergum OR aspex OR aspisol OR asteric OR astrix OR bamyl OR biprin OR boxazin OR caprin OR cardioaspirina OR cartia OR caspirin OR catalgine OR catalgix OR claradin OR colfarit OR contrheuma OR dispirin OR dolean OR durlaza OR dusil OR easprin OR ecasil OR ecosprin OR ecotrin OR empirin OR endosprin OR entericin OR enterosarine OR entrophen OR extren OR globentyl OR godamed OR helicon OR idotyl OR istopirin OR magnecyl OR measurin OR melabon OR micristin OR mikristin OR naspro OR "nu-seal*" OR nuseals OR ostoprin OR paracin OR pengo OR polopiryna OR polopirin OR premaspin OR reumyl OR rhodine OR rhonal OR ronal OR salacetin OR saletin OR sedergine OR "sodium acetylsalicylate" OR solpyron OR solprin OR solupsa* OR tapal OR temagin OR tromalyt OR turivital OR vazalore OR verin OR vitalink OR xaxa OR zorprin OR ajoene OR aloxiprin OR anagrelide OR ancrod OR applaggin OR aprosulate OR aspalatone OR ataprost OR atopaxar OR beraprost OR brilinta OR brilique OR cangrelor OR caplacizumab OR cilostazol OR clopidogrel OR clopilet OR cicaprost OR ciprostene OR cryptolepine OR dazoxiben OR dehydrocilostazol OR dermatan OR dextran* OR dipyridamole OR elinogrel OR "enfenamic acid" OR esuberaprost OR glenzocimab OR ghilanten OR ibustrin OR iloprost* OR ifetroban OR imolamine OR indobufen OR isbogrel OR iscover OR itazigrel OR linotroban OR lixazinone OR nafazatrom OR naxaprostene OR octimibate OR oxagrelate OR pamicogrel OR "pentosan polysulfate*" OR picotamide OR piracetam OR plafibride OR plavix OR possia OR prasugrel OR prostacyclin OR pentoxifyllin* OR prostacyclin OR prasugrel* OR rafigrelide OR samixogrel OR sarpogrelate OR satigrel OR selatogrel OR sulfinpyrazone OR taprostene OR terbogrel OR terutroban OR triflusal OR ticagrelor OR ticlopidine OR tretoquinol OR uproleselan OR abciximab OR aggrastat OR albolabrin OR arginylglycylaspartylserine OR bitistatin OR contortrostatin OR disintegrin* OR echistatin OR elarofiban OR eptifibatid* OR fradafiban OR gantofiban OR glycylarginylglycylaspartylserine OR integrilin AND kistrin OR lamifiban OR lefradafiban OR lotrafiban OR orbofiban OR "repro" OR roxifiban OR sibrafiban OR tirofiban OR triflavin OR trigamin OR xemilofiban OR annexin OR caplacizumab OR effient OR efient OR prasugrel ) ) AND (TITLE-ABS-KEY ("Acute Coronary Syndrome" OR "Myocardial Infarction" OR "Non-ST Elevated Myocardial Infarction" OR "ST Elevation Myocardial Infarction" OR "Angina, Unstable" OR "Coronary Artery Disease" OR "Coronary Stenosis" OR "Coronary Disease" OR "cardiac infarct" OR "cardiac infarction" OR "cardial infarct" OR "heart attack" OR "heart infarct" OR "heart infarction" OR "heart micro infarction" OR "heart muscle infarction" OR "infarction, heart" OR "myocardial infarct" OR "myocardial infarction" OR "myocardium infarct" OR "myocardium infarction" OR "second heart attack" OR "subendocardial infarction" OR "transmural cardiac infarction" OR "transmural heart infarction" OR "transmural infarction, heart" OR "acute coronary syndrome" OR "acute coronary syndromes" OR acs OR ami OR "st elevated mi" OR "st elevated myocardial infarction" OR "st elevation mi" OR "stelevation myocardial infarction" OR "st segment elevated myocardial infarction" OR "st segment elevation mi" OR "st segment elevation heart infarction" OR "st segment elevation myocardial infarction" OR stemi OR nstemi OR "non st elevated mi" OR "non st elevated myocardial infarction" OR "non st elevation mi" OR "non st elevation myocardial infarction" OR "non st segment elevated myocardial infarction" OR "non st segment elevation mi" OR "non st segment elevation heart infarction" OR "non stsegment elevation myocardial infarction" OR "non stemi" OR "non-st elevated myocardial infarction" OR "angina pectoris, unstable" OR "angina, unstable" OR "unstable angina" OR "unstable angina pectoris" OR uap )) AND (TITLE-ABS-KEY ("Adult" OR adult* OR man OR men OR woman OR women )) | 188 |

| Table S2. Patient history of underlying diseases information | | | | | | | | |
| --- | --- | --- | --- | --- | --- | --- | --- | --- |
| Study | Hypertension | Diabetes mellitus | Dyslipidemia | Smoking | Family history | Chronic renal failure | Prior myocardial infarction | Prior stroke |
| Djohan2022 | 918 | 1552 | 1078 | 1073 | 208 | 99 | 204 | 104 |
| Doi2017 | 1124 | 667 | 920 | 1207 | - | - | 1361 | - |
| Gunasekaran2019 | 286 | 164 | - | 229 | - | 97 | - | - |
| Joo2018 | 197 | 93 | 169 | 96 | - | - | 21 | 16 |
| Liang2019 | 77 | 36 | - | 63 | 48 | - | 16 | - |
| Núñez-Gil2018 | 163 | 73 | 165 | 171 | 13 | 22 | 46 | - |
| Shanmugam2017 | 43 | 21 | 40 | 74 | 27 | 15 | 6 | 1 |

| Table S3. Artery involvement information | | | | | | | |
| --- | --- | --- | --- | --- | --- | --- | --- |
| Study | Single vessel disease | Stenosis | | | Culprit artery | | |
|  |  | LAD | LCx | RCA | LAD | LCx | RCA |
| Djohan2022 | - | 1230 | 442 | 921 | 850 | 212 | 695 |
| Doi2017 | 22 | - | - | - | 22 | 28 | 39 |
| Gunasekaran2019 | 148 |  |  |  | 149 | 96 | 185 |
| Joo2018 |  | - | - | - | 283 | 40 | 24 |
| Liang2019 | 6 | 101 | 94 | 99 | 42 | 44 | 76 |
| Núñez-Gil2018 | 80 | - | - | - | - | - | - |
| Shanmugam2017 | 60 | - | - | - | 42 | 9 | 55 |


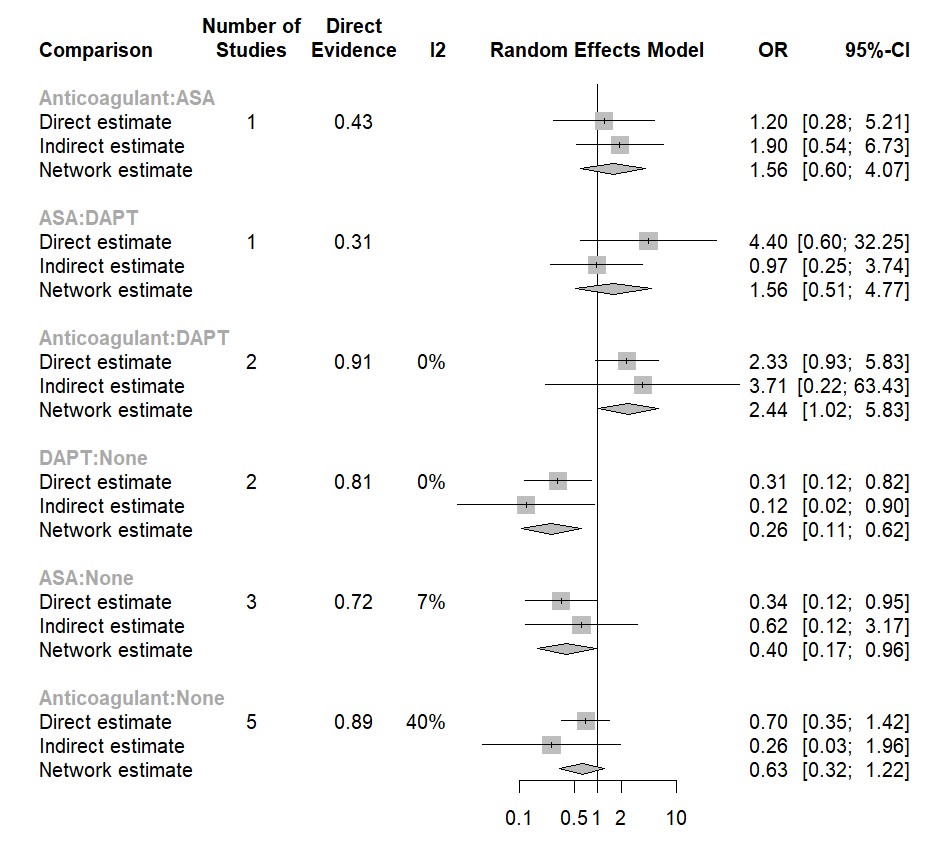


Figure S1. Network Split for direct, indirect, and network estimates


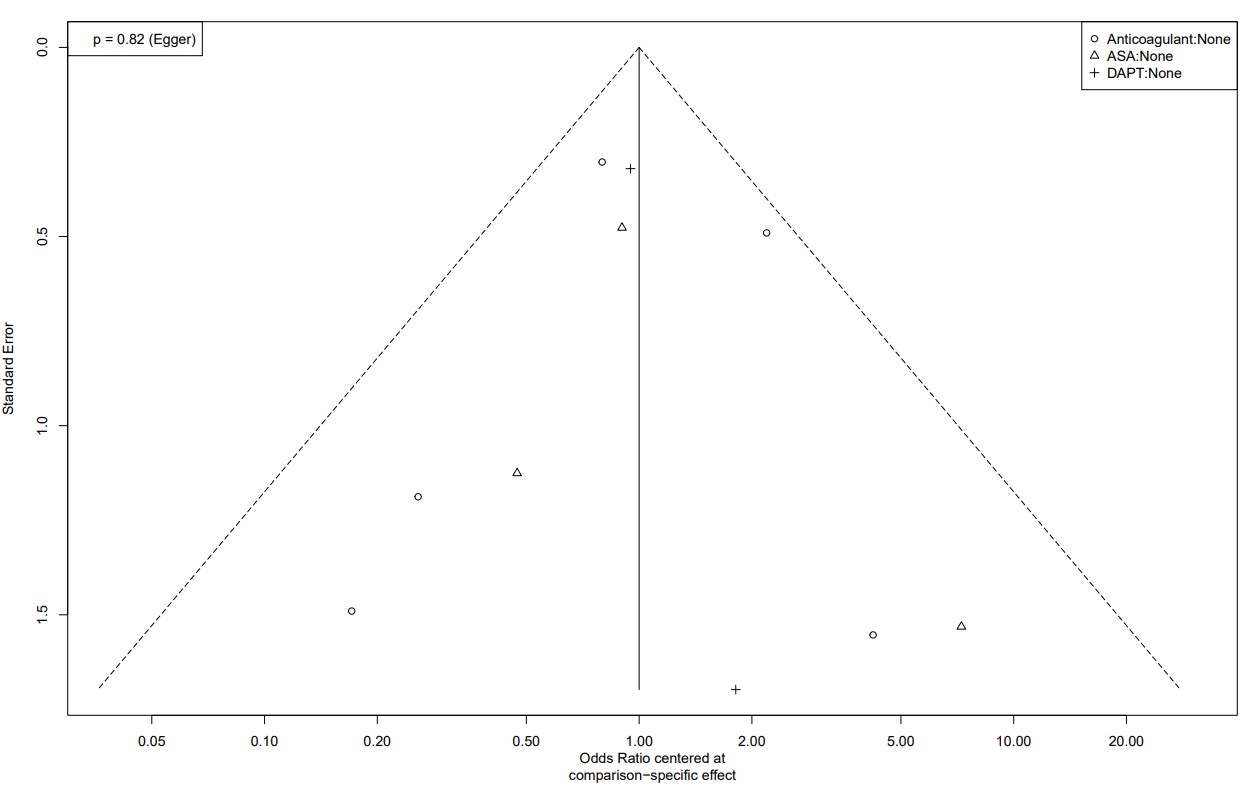


Figure S2. Network funnel plot of eligible comparisons for MACE
